# 4-Chloro-*N*-(3-chloro­benzo­yl)benzene­sulfonamide monohydrate

**DOI:** 10.1107/S1600536810023962

**Published:** 2010-06-26

**Authors:** P. A. Suchetan, B. Thimme Gowda, Sabine Foro, Hartmut Fuess

**Affiliations:** a Department of Chemistry, Mangalore University, Mangalagangotri 574 199, Mangalore, India; b Institute of Materials Science, Darmstadt University of Technology, Petersenstrasse 23, D-64287 Darmstadt, Germany

## Abstract

In the title compound, C_13_H_9_Cl_2_NO_3_S·H_2_O, the conformation of the C=O bond is *syn* to the *meta*-Cl group in the benzoyl ring. The mol­ecules are twisted at the S—N bond with a C—S—N—C torsion angle of 72.9 (2)°. The dihedral angle between the sulfonyl benzene ring and the S—NH—C—O segment is 77.8 (1)° and that between the sulfonyl and benzoyl benzene rings is 80.5 (1)°. In the crystal, mol­ecules are linked into a two-dimensional network parallel to (100) by N—H⋯O and O—H⋯O hydrogen bonds.

## Related literature

For background to our study of the effect of ring and side-chain substituents on the crystal structures of *N*-aromatic sulfonamides and for related structures, see: Gowda *et al.* (2009 ▶, 2010 ▶); Suchetan *et al.* (2010**a* ▶,b* ▶).
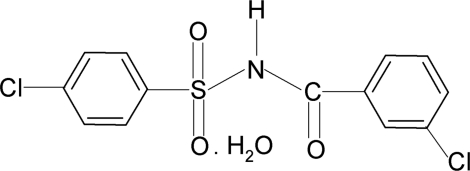


## Experimental

### 

#### Crystal data

C_13_H_9_Cl_2_NO_3_S·H_2_O
*M* *_r_* = 348.19
Monoclinic, 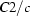

*a* = 46.909 (3) Å
*b* = 4.9469 (5) Å
*c* = 12.919 (1) Å
β = 95.938 (9)°
*V* = 2981.8 (4) Å^3^
*Z* = 8
Mo *K*α radiation
μ = 0.59 mm^−1^
*T* = 299 K
0.30 × 0.14 × 0.10 mm

#### Data collection

Oxford Diffraction Xcalibur diffractometer with a Sapphire CCD detector
Absorption correction: multi-scan (*CrysAlis RED*; Oxford Diffraction, 2009 ▶) *T*_min_ = 0.843, *T*_max_ = 0.944
7848 measured reflections
2511 independent reflections
2027 reflections with *I* > 2σ(*I*)
*R*_int_ = 0.094

#### Refinement

*R*[*F* ^2^ > 2σ(*F* ^2^)] = 0.066
*wR*(*F* ^2^) = 0.184
*S* = 1.01
2511 reflections
199 parameters
3 restraints
H atoms treated by a mixture of independent and constrained refinement
Δρ_max_ = 0.49 e Å^−3^
Δρ_min_ = −0.48 e Å^−3^

### 

Data collection: *CrysAlis CCD* (Oxford Diffraction, 2009 ▶); cell refinement: *CrysAlis RED* (Oxford Diffraction, 2009 ▶); data reduction: *CrysAlis RED*; program(s) used to solve structure: *SHELXS97* (Sheldrick, 2008 ▶); program(s) used to refine structure: *SHELXL97* (Sheldrick, 2008 ▶); molecular graphics: *PLATON* (Spek, 2009 ▶); software used to prepare material for publication: *SHELXL97*.

## Supplementary Material

Crystal structure: contains datablocks I, global. DOI: 10.1107/S1600536810023962/ci5109sup1.cif

Structure factors: contains datablocks I. DOI: 10.1107/S1600536810023962/ci5109Isup2.hkl

Additional supplementary materials:  crystallographic information; 3D view; checkCIF report

## Figures and Tables

**Table 1:** Hydrogen-bond geometry (Å, °)

*D*—H⋯*A*	*D*—H	H⋯*A*	*D*⋯*A*	*D*—H⋯*A*
N1—H1*N*⋯O4^i^	0.83 (2)	1.98 (2)	2.805 (4)	175 (3)
O4—H41⋯O2^ii^	0.83 (2)	2.17 (3)	2.944 (3)	154 (4)
O4—H42⋯O1	0.85 (2)	2.32 (4)	3.035 (4)	142 (5)
O4—H42⋯O3	0.85 (2)	2.27 (4)	2.952 (3)	137 (5)

Symmetry codes: (i) 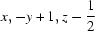; (ii) 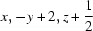.

**Fig. 1.**
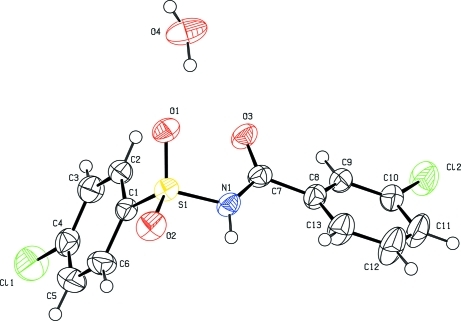
Molecular structure of the title compound, showing the atom-labelling scheme. Displacement ellipsoids are drawn at the 50% probability level.

**Fig. 2.**
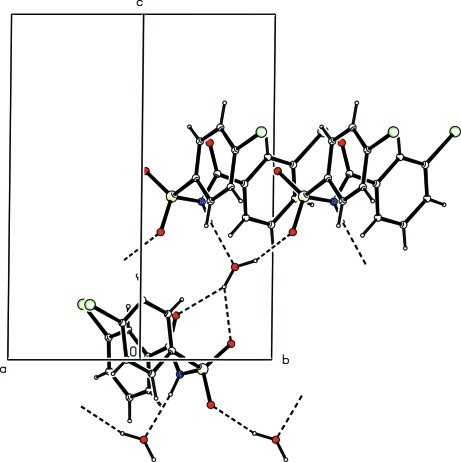
Part of the crystal packing in the title compound, showing hydrogen bonds (dashed lines) involving the water molecules.

## supplementary crystallographic information

### Comment

As a part of studying the effect of ring and the side chain substituents on the crystal structures of *N*-aromatic sulfonamides (Gowda *et al.*, 2009, 2010; Suchetan *et al.*, 2010*a,b*), the structure of 4-chloro-*N*-(3-Chlorobenzoyl)benzenesulfonamide monohydrate (I) has been determined (Fig.1).

The conformation of the N—H bond in the C—SO_2_—NH—C(O) segment is *anti* to the C═O bond, similar to those observed in *N*-(3-chlorobenzoyl)-benzenesulfonamide (II) (Gowda *et al.*, 2009), *N*-(benzoyl)-4-chlorobenzenesulfonamide (III) (Suchetan *et al.*, 2010*a*), 4-chloro-*N*-(2-chlorobenzoyl)benzenesulfonamide (IV) (Gowda *et al.*, 2010), and *N*-(4-chlorobenzoyl)-4- chlorobenzenesulfonamide (V) (Suchetan *et al.*, 2010*b*).

Further, the conformation of the C═O bond in the C—SO_2_—NH—C(O) segment of (I) is *syn* to the *meta*-Cl in the benzoyl ring, similar to that observed between the C═O bond and *ortho*-Cl in (IV), but contrary to the *anti* conformation observed between the C═O bond and *meta*-Cl in (II).

The molecules are twisted at the S—N bond with the C1—S1—N1—C7 torsional angle of 72.9 (2)°, compared to those of 65.3 (2)° in (II), -70.0 (2)°, 61.3 (2)° in the two independent molecules of (III), 65.7 (2)° in (IV) and 67.5 (3)° in (V).

The dihedral angles between the sulfonyl benzene ring and the S/N/C/O plane is 77.8 (1)°, compared to the values of 89.9 (1)° in (II), 72.0 (1)° & 77.3 (1)° in the two molecules of (III), 88.5 (1)° in (IV) and 79.0 (1)° in (V).

Furthermore, the dihedral angle between the sulfonyl and the benzoyl benzene rings is 80.5 (1)°, compared to the values of 87.5 (1)° in (II), 62.8 (1)° (molecule 1) and 78.6 (1)° (molecule 2) of (III), 58.0 (1)° in (IV) and 85.6 (1)° in (V)

The molecules are linked into a two-dimensional network (Fig.2) parallel to the (100) by N—H···O and O—H···O hydrogen bonds (Table 1).

### Experimental

The title compound was prepared by refluxing a mixture of 3-chlorobenzoic acid, 4-chlorobenzenesulfonamide and phosphorous oxy chloride for 3 h on a water bath. The resultant mixture was cooled and poured into ice cold water. The solid obtained was filtered, washed thoroughly with water and then dissolved in sodium bicarbonate solution. The compound was later reprecipitated by acidifying the filtered solution with dilute HCl. It was filtered, dried and recrystallized. Long needle like colourless single crystals of the title compound used in X-ray diffraction studies were obtained by slow evaporation of its toluene solution at room temperature.

### Refinement

The H atom of the NH group and the H atoms of the water molecule were located in a difference map and later restrained to N–H = 0.86 (2) %A and to O–H = 0.85 (2) %A. The other H atoms were positioned with idealized geometry using a riding model with C–H = 0.93 Å. All H atoms were refined with isotropic displacement parameters (set to 1.2 times of the *U*_eq_ of the parent atom).

### Crystal data

**Table:**

C_13_H_9_Cl_2_NO_3_S·H_2_O	*F*(000) = 1424
*M**_r_* = 348.19	*D*_x_ = 1.551 Mg m^−^^3^
Monoclinic, *C*2/*c*	Mo *K*α radiation, λ = 0.71073 Å
Hall symbol: -C 2yc	Cell parameters from 3786 reflections
*a* = 46.909 (3) Å	θ = 27.9–2.6°
*b* = 4.9469 (5) Å	µ = 0.59 mm^−^^1^
*c* = 12.919 (1) Å	*T* = 299 K
β = 95.938 (9)°	Long needle, colourless
*V* = 2981.8 (4) Å^3^	0.30 × 0.14 × 0.10 mm
*Z* = 8	

### Data collection

**Table:**

Oxford Diffraction Xcalibur diffractometer with a Sapphire CCD detector	2511 independent reflections
Radiation source: fine-focus sealed tube	2027 reflections with *I* > 2σ(*I*)
graphite	*R*_int_ = 0.094
Rotation method data acquisition using ω and φ scans	θ_max_ = 25.4°, θ_min_ = 2.6°
Absorption correction: multi-scan (*CrysAlis RED*; Oxford Diffraction, 2009)	*h* = −56→55
*T*_min_ = 0.843, *T*_max_ = 0.944	*k* = −5→5
7848 measured reflections	*l* = −15→15

### Refinement

**Table:**

Refinement on *F*^2^	Primary atom site location: structure-invariant direct methods
Least-squares matrix: full	Secondary atom site location: difference Fourier map
*R*[*F*^2^ > 2σ(*F*^2^)] = 0.066	Hydrogen site location: inferred from neighbouring sites
*wR*(*F*^2^) = 0.184	H atoms treated by a mixture of independent and constrained refinement
*S* = 1.01	*w* = 1/[σ^2^(*F*_o_^2^) + (0.1432*P*)^2^] where *P* = (*F*_o_^2^ + 2*F*_c_^2^)/3
2511 reflections	(Δ/σ)_max_ = 0.008
199 parameters	Δρ_max_ = 0.49 e Å^−^^3^
3 restraints	Δρ_min_ = −0.48 e Å^−^^3^

### Special details

**Table:**

Geometry. All e.s.d.'s (except the e.s.d. in the dihedral angle between two l.s. planes) are estimated using the full covariance matrix. The cell e.s.d.'s are taken into account individually in the estimation of e.s.d.'s in distances, angles and torsion angles; correlations between e.s.d.'s in cell parameters are only used when they are defined by crystal symmetry. An approximate (isotropic) treatment of cell e.s.d.'s is used for estimating e.s.d.'s involving l.s. planes.
Refinement. Refinement of *F*^2^ against ALL reflections. The weighted *R*-factor *wR* and goodness of fit *S* are based on *F*^2^, conventional *R*-factors *R* are based on *F*, with *F* set to zero for negative *F*^2^. The threshold expression of *F*^2^ > σ(*F*^2^) is used only for calculating *R*-factors(gt) *etc*. and is not relevant to the choice of reflections for refinement. *R*-factors based on *F*^2^ are statistically about twice as large as those based on *F*, and *R*- factors based on ALL data will be even larger.

### Fractional atomic coordinates and isotropic or equivalent isotropic displacement parameters (Å^2^)

**Table:**

	*x*	*y*	*z*	*U*_iso_*/*U*_eq_	
Cl1	0.23824 (2)	−0.1411 (2)	0.15914 (10)	0.0751 (4)	
Cl2	0.030813 (19)	−0.4014 (2)	0.16022 (8)	0.0636 (4)	
S1	0.145674 (14)	0.62369 (12)	−0.02750 (5)	0.0330 (3)	
O1	0.13859 (4)	0.8302 (4)	0.04597 (17)	0.0413 (5)	
O2	0.15376 (5)	0.6968 (4)	−0.13059 (16)	0.0478 (6)	
O3	0.10727 (4)	0.3795 (4)	0.12809 (15)	0.0433 (6)	
N1	0.11762 (5)	0.4252 (5)	−0.04251 (18)	0.0348 (6)	
H1N	0.1192 (7)	0.357 (6)	−0.1005 (17)	0.042*	
C1	0.17257 (6)	0.4165 (5)	0.0259 (2)	0.0332 (6)	
C2	0.17479 (6)	0.3855 (6)	0.1325 (2)	0.0398 (7)	
H2	0.1630	0.4779	0.1740	0.048*	
C3	0.19519 (7)	0.2122 (7)	0.1723 (2)	0.0456 (7)	
H3	0.1976	0.1796	0.2436	0.055*	
C4	0.21297 (6)	0.0798 (6)	0.1065 (3)	0.0453 (8)	
C5	0.21165 (7)	0.1130 (7)	0.0002 (3)	0.0544 (9)	
H5	0.2240	0.0242	−0.0404	0.065*	
C6	0.19104 (7)	0.2844 (7)	−0.0406 (2)	0.0476 (8)	
H6	0.1888	0.3170	−0.1119	0.057*	
C7	0.10191 (6)	0.3242 (5)	0.0382 (2)	0.0340 (6)	
C8	0.07754 (6)	0.1437 (5)	0.0091 (2)	0.0363 (7)	
C9	0.06672 (5)	−0.0221 (6)	0.0866 (2)	0.0375 (6)	
H9	0.0754	−0.0138	0.1546	0.045*	
C10	0.04422 (6)	−0.1911 (6)	0.0642 (3)	0.0428 (7)	
C11	0.03190 (7)	−0.1960 (8)	−0.0333 (3)	0.0600 (10)	
H11	0.0163	−0.3092	−0.0514	0.072*	
C12	0.04251 (8)	−0.0290 (9)	−0.1094 (3)	0.0681 (11)	
H12	0.0335	−0.0357	−0.1770	0.082*	
C13	0.06525 (7)	0.1418 (7)	−0.0887 (3)	0.0516 (9)	
H13	0.0718	0.2507	−0.1399	0.062*	
O4	0.12309 (7)	0.8386 (5)	0.26833 (19)	0.0640 (7)	
H41	0.1273 (10)	0.997 (5)	0.287 (4)	0.096*	
H42	0.1245 (11)	0.760 (10)	0.211 (2)	0.096*	

### Atomic displacement parameters (Å^2^)

**Table:**

	*U*^11^	*U*^22^	*U*^33^	*U*^12^	*U*^13^	*U*^23^
Cl1	0.0623 (6)	0.0664 (7)	0.0913 (9)	0.0265 (4)	−0.0185 (6)	−0.0069 (5)
Cl2	0.0642 (6)	0.0712 (6)	0.0563 (6)	−0.0273 (4)	0.0111 (5)	0.0027 (4)
S1	0.0396 (4)	0.0302 (4)	0.0291 (4)	−0.0026 (2)	0.0029 (3)	0.0016 (2)
O1	0.0499 (11)	0.0297 (10)	0.0434 (11)	−0.0007 (8)	0.0003 (9)	−0.0043 (9)
O2	0.0608 (13)	0.0483 (12)	0.0340 (11)	−0.0061 (10)	0.0041 (10)	0.0097 (10)
O3	0.0485 (11)	0.0524 (13)	0.0282 (11)	−0.0123 (9)	0.0004 (9)	−0.0059 (8)
N1	0.0377 (12)	0.0387 (12)	0.0269 (12)	−0.0045 (10)	−0.0023 (10)	−0.0021 (10)
C1	0.0328 (13)	0.0335 (13)	0.0335 (15)	−0.0047 (11)	0.0042 (11)	−0.0027 (11)
C2	0.0350 (14)	0.0480 (16)	0.0357 (16)	0.0033 (12)	0.0011 (12)	−0.0058 (12)
C3	0.0426 (15)	0.0536 (17)	0.0388 (15)	0.0045 (14)	−0.0035 (13)	−0.0015 (15)
C4	0.0371 (15)	0.0411 (15)	0.055 (2)	0.0041 (12)	−0.0075 (14)	−0.0033 (14)
C5	0.0468 (17)	0.060 (2)	0.057 (2)	0.0109 (15)	0.0100 (16)	−0.0134 (16)
C6	0.0504 (17)	0.0571 (18)	0.0363 (15)	0.0051 (15)	0.0089 (13)	−0.0062 (15)
C7	0.0363 (13)	0.0347 (13)	0.0301 (14)	0.0027 (11)	−0.0017 (11)	−0.0032 (11)
C8	0.0324 (13)	0.0362 (14)	0.0388 (15)	0.0003 (11)	−0.0035 (12)	−0.0020 (11)
C9	0.0337 (14)	0.0455 (16)	0.0326 (14)	−0.0021 (12)	−0.0003 (12)	−0.0046 (12)
C10	0.0372 (15)	0.0464 (16)	0.0450 (17)	−0.0074 (12)	0.0051 (13)	−0.0039 (14)
C11	0.0472 (18)	0.075 (2)	0.054 (2)	−0.0219 (17)	−0.0127 (16)	−0.0065 (19)
C12	0.061 (2)	0.090 (3)	0.047 (2)	−0.023 (2)	−0.0225 (18)	0.005 (2)
C13	0.0528 (18)	0.062 (2)	0.0375 (17)	−0.0116 (15)	−0.0075 (15)	0.0068 (14)
O4	0.111 (2)	0.0504 (14)	0.0298 (12)	0.0019 (14)	0.0024 (13)	0.0003 (11)

### Geometric parameters (Å, °)

**Table:**

Cl1—C4	1.701 (3)	C5—C6	1.351 (4)
Cl2—C10	1.782 (3)	C5—H5	0.93
S1—O1	1.456 (2)	C6—H6	0.93
S1—O2	1.467 (2)	C7—C8	1.468 (4)
S1—N1	1.637 (2)	C8—C13	1.333 (4)
S1—C1	1.714 (3)	C8—C9	1.427 (4)
O3—C7	1.195 (3)	C9—C10	1.354 (4)
N1—C7	1.428 (4)	C9—H9	0.93
N1—H1N	0.833 (18)	C10—C11	1.331 (5)
C1—C2	1.379 (4)	C11—C12	1.413 (6)
C1—C6	1.438 (4)	C11—H11	0.93
C2—C3	1.347 (4)	C12—C13	1.365 (5)
C2—H2	0.93	C12—H12	0.93
C3—C4	1.412 (5)	C13—H13	0.93
C3—H3	0.93	O4—H41	0.83 (2)
C4—C5	1.379 (5)	O4—H42	0.85 (2)
			
O1—S1—O2	121.10 (13)	C5—C6—H6	119.9
O1—S1—N1	105.25 (13)	C1—C6—H6	119.9
O2—S1—N1	108.69 (12)	O3—C7—N1	123.9 (2)
O1—S1—C1	111.44 (13)	O3—C7—C8	117.9 (3)
O2—S1—C1	105.30 (14)	N1—C7—C8	118.2 (2)
N1—S1—C1	103.79 (12)	C13—C8—C9	120.7 (3)
C7—N1—S1	126.36 (17)	C13—C8—C7	119.7 (3)
C7—N1—H1N	128 (2)	C9—C8—C7	119.6 (2)
S1—N1—H1N	102 (2)	C10—C9—C8	122.1 (3)
C2—C1—C6	123.9 (3)	C10—C9—H9	119.0
C2—C1—S1	116.4 (2)	C8—C9—H9	119.0
C6—C1—S1	119.7 (2)	C11—C10—C9	117.8 (3)
C3—C2—C1	115.5 (3)	C11—C10—Cl2	119.9 (2)
C3—C2—H2	122.3	C9—C10—Cl2	122.4 (2)
C1—C2—H2	122.3	C10—C11—C12	119.9 (3)
C2—C3—C4	120.4 (3)	C10—C11—H11	120.1
C2—C3—H3	119.8	C12—C11—H11	120.1
C4—C3—H3	119.8	C13—C12—C11	123.3 (3)
C5—C4—C3	125.2 (3)	C13—C12—H12	118.3
C5—C4—Cl1	115.6 (3)	C11—C12—H12	118.3
C3—C4—Cl1	119.2 (3)	C8—C13—C12	116.3 (3)
C6—C5—C4	114.8 (3)	C8—C13—H13	121.9
C6—C5—H5	122.6	C12—C13—H13	121.9
C4—C5—H5	122.6	H41—O4—H42	130 (5)
C5—C6—C1	120.2 (3)		
			
O1—S1—N1—C7	−44.3 (2)	S1—C1—C6—C5	−177.6 (3)
O2—S1—N1—C7	−175.4 (2)	S1—N1—C7—O3	1.7 (4)
C1—S1—N1—C7	72.9 (2)	S1—N1—C7—C8	−178.91 (19)
O1—S1—C1—C2	27.9 (3)	O3—C7—C8—C13	159.3 (3)
O2—S1—C1—C2	161.0 (2)	N1—C7—C8—C13	−20.1 (4)
N1—S1—C1—C2	−84.9 (2)	O3—C7—C8—C9	−18.9 (4)
O1—S1—C1—C6	−152.8 (2)	N1—C7—C8—C9	161.7 (2)
O2—S1—C1—C6	−19.7 (3)	C13—C8—C9—C10	1.5 (5)
N1—S1—C1—C6	94.4 (2)	C7—C8—C9—C10	179.7 (3)
C6—C1—C2—C3	−2.2 (4)	C8—C9—C10—C11	−1.0 (5)
S1—C1—C2—C3	177.0 (2)	C8—C9—C10—Cl2	179.3 (2)
C1—C2—C3—C4	1.1 (4)	C9—C10—C11—C12	0.3 (6)
C2—C3—C4—C5	0.5 (5)	Cl2—C10—C11—C12	179.9 (3)
C2—C3—C4—Cl1	−179.2 (2)	C10—C11—C12—C13	0.1 (7)
C3—C4—C5—C6	−1.1 (5)	C9—C8—C13—C12	−1.1 (5)
Cl1—C4—C5—C6	178.6 (2)	C7—C8—C13—C12	−179.3 (3)
C4—C5—C6—C1	0.1 (5)	C11—C12—C13—C8	0.4 (6)
C2—C1—C6—C5	1.6 (5)		

### Hydrogen-bond geometry (Å, °)

**Table:**

*D*—H···*A*	*D*—H	H···*A*	*D*···*A*	*D*—H···*A*
N1—H1N···O4^i^	0.83 (2)	1.98 (2)	2.805 (4)	175 (3)
O4—H41···O2^ii^	0.83 (2)	2.17 (3)	2.944 (3)	154 (4)
O4—H42···O1	0.85 (2)	2.32 (4)	3.035 (4)	142 (5)
O4—H42···O3	0.85 (2)	2.27 (4)	2.952 (3)	137 (5)

Symmetry codes: (i) *x*, −*y*+1, *z*−1/2; (ii) *x*, −*y*+2, *z*+1/2.
